# PHB is Produced from Glycogen Turn-over during Nitrogen Starvation in *Synechocystis* sp. PCC 6803

**DOI:** 10.3390/ijms20081942

**Published:** 2019-04-20

**Authors:** Moritz Koch, Sofía Doello, Kirstin Gutekunst, Karl Forchhammer

**Affiliations:** 1 Interfaculty Institute of Microbiology and Infection Medicine Tübingen, Eberhard-Karls-Universität Tübingen, 72076 Tübingen, Germany; moritz.koch@uni-tuebingen.de (M.K.); sofia.doello@gmail.com (S.D.); 2 Department of Biology, Botanical Institute, Christian-Albrechts-University, 24118 Kiel, Germany; kgutekunst@bot.uni-kiel.de

**Keywords:** cyanobacteria, bioplastic, PHB, sustainable, glycogen, metabolic engineering, Synechocystis

## Abstract

Polyhydroxybutyrate (PHB) is a polymer of great interest as a substitute for conventional plastics, which are becoming an enormous environmental problem. PHB can be produced directly from CO_2_ in photoautotrophic cyanobacteria. The model cyanobacterium *Synechocystis* sp. PCC 6803 produces PHB under conditions of nitrogen starvation. However, it is so far unclear which metabolic pathways provide the precursor molecules for PHB synthesis during nitrogen starvation. In this study, we investigated if PHB could be derived from the main intracellular carbon pool, glycogen. A mutant of the major glycogen phosphorylase, GlgP2 (*slr1367* product), was almost completely impaired in PHB synthesis. Conversely, in the absence of glycogen synthase GlgA1 (*sll0945* product), cells not only produced less PHB, but were also impaired in acclimation to nitrogen depletion. To analyze the role of the various carbon catabolic pathways (EMP, ED and OPP pathways) for PHB production, mutants of key enzymes of these pathways were analyzed, showing different impact on PHB synthesis. Together, this study clearly indicates that PHB in glycogen-producing *Synechocystis* sp. PCC 6803 cells is produced from this carbon-pool during nitrogen starvation periods. This knowledge can be used for metabolic engineering to get closer to the overall goal of a sustainable, carbon-neutral bioplastic production.

## 1. Introduction

Cyanobacteria are among the most widespread organisms on our planet. Their ability to perform oxygenic photosynthesis allows them to grow autotrophically with CO_2_ as the sole carbon source [1]. Additionally, many cyanobacteria acquired the ability to fix nitrogen, one of the most limiting nutrients [2]. However, many others are not able to fix nitrogen, one of them being the well-studied model organism *Synechocystis* sp. PCC 6803 (hereafter: *Synechocystis*) [3]. Nitrogen starvation starts a well-orchestrated survival process in *Synechocystis*, called chlorosis [4]. During chlorosis, *Synechocystis* degrades not only its photosynthetic machinery, but also accumulates large quantities of biopolymers, namely glycogen and poly-hydroxy-butyrate (PHB) [5]. Glycogen synthesis following the onset of nitrogen starvation serves transiently as a major sink for newly fixed CO_2_ [6] before CO_2_ fixation is tuned down during prolonged nitrogen starvation. During resuscitation from chlorosis, a specific glycogen catabolic metabolism supports the re-greening of chlorotic cells [7]. By contrast to the pivotal role of glycogen, the function of the polymer PHB remains puzzling, since mutants impaired in PHB synthesis survived and recovered from chlorosis as awild-type [8,9]. Nevertheless, many different cyanobacterial species produce PHB, implying a hitherto unrecognized functional importance [10]. In other microorganisms PHB fulfills various functions during conditions of unbalanced nutrient availability and can also protect cells against low temperatures or redox stress [11,12,13]. Understanding the intracellular mechanisms that lead to PHB production could help to elucidate the physiological role of this polymer. Regardless of the physiological significance of PHB, this polymer has been recognized as a promising alternative for current plastics, which contaminate terrestrial and aquatic ecosystems [14]. PHB can serve as a basis for completely biodegradable plastics, with properties comparable to petroleum-derived plastics [15,16]. Since *Synechocystis* produces PHB only under nutrient limiting conditions, this phenomenon can be exploited to temporally separate the initial biomass production from PHB production induced by shifting cells to nitrogen limiting conditions [10].

One of the biggest obstacles preventing economic PHB production in cyanobacteria remains the low level of intracellular PHB accumulation [17]. While chemotrophic bacteria are capable of producing more than 80% PHB of their cell dry mass, (e.g., *Cupriavidus necator*), most cyanobacteria naturally produce less than 20% of their cell dry mass [15]. Additionally, their growth rate is too slow to compete with the PHB production in chemotrophic bacteria. There have been many attempts in the past to further improve the intracellular PHB production, often with limited [1] success [18,19,20]. One of the most successful approaches has been achieved by random mutagenesis, leading to up to 37% PHB of the cell dry mass [21]. However, more directed approaches involving genetic engineering are often limited by a lack of knowledge about how the cells’ metabolism works in detail. For example, until today, it was still unknown from which carbon metabolites PHB was derived. There have been several different studies analyzing the intracellular fluxes in cyanobacteria [22]. However, most of them did not analyze the carbon flow during prolonged nitrogen starvation. One of these studies showed that in nitrogen-starved photosynthetically grown cyanobacteria up to 87% of the carbon in PHB is derived from intracellular carbon sources rather than from newly fixed CO_2_ [23]. However, until now, it was not clearly resolved which metabolic routes provide the precursors for PHB synthesis. This knowledge would lay the foundation for future metabolic engineering approaches to create overproduction strains. Hence, the goal of this study was to find out where the carbon for the PHB production is coming from and which pathways it is taking until it reaches PHB.

It has been shown that disruption of PHB synthesis results in an increased production of glycogen; however, an overproduction of glycogen did not lead to higher amounts of PHB [24]. Another study that also investigated the accumulation of glycogen in a PHB-free mutant ∆*phaC*, could not detect any differences in growth or glycogen accumulation [8].

**Figure 1:**
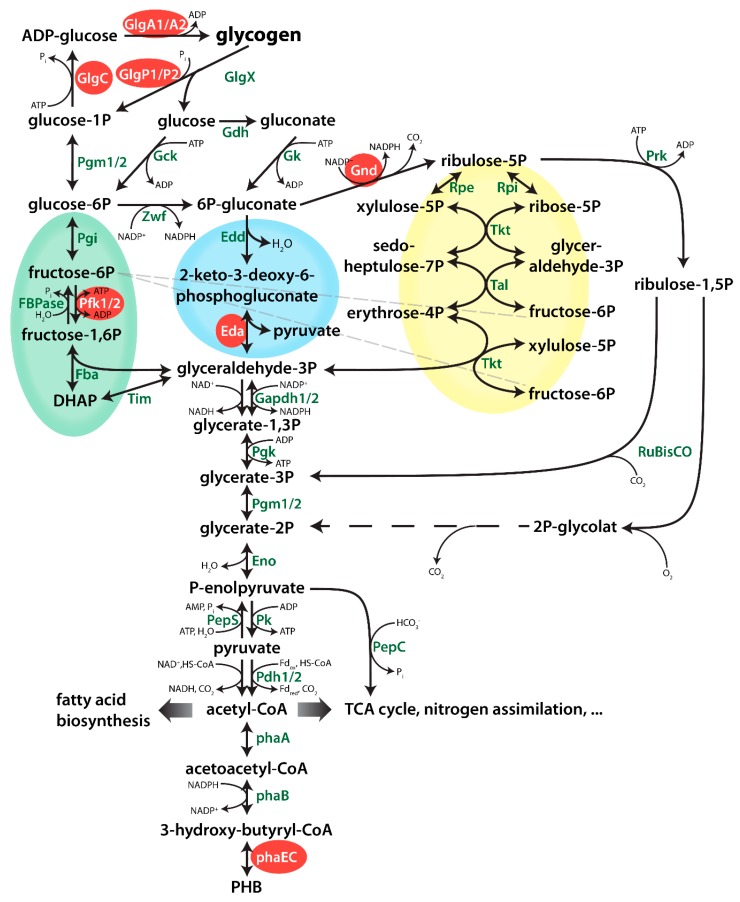
Overview of central metabolism of Synechocystis. Genes which were deleted in this study are highlighted in with a red background. Dotted lines represent several enzymatic reactions. The EMP, ED and OPP (Embden-Meyerhof-Parnas, Entner-Doudoroff, Oxidative Pentose Phosphate) pathways are highlighted in green, blue and yellow, respectively.

An important aspect in the issue concerning the relation between glycogen and PHB metabolism deals with the contribution of various carbon metabolic pathways for the production of precursors for PHB under conditions of nitrogen limitation. *Synechocystis* is able to catabolize glucose via three parallel operating glycolytic pathways [25] (Figure 1): the Embden-Meyerhof-Parnas (EMP) pathway, the oxidative pentose phosphate (OPP) pathway [26], and the Entner Doudoroff (ED) pathway [25]. When nitrogen-starved cells recover from chlorosis, they require the parallel operating OPP and ED pathways, whereas the EMP pathway seems dispensable [7]. Metabolic analysis of mutants overexpressing the transcriptional regulator *rre37* showed a correlated upregulation of PHB synthesis and EMP pathway genes (*phaAB* and *pfkA*, respectively) [27]. However, so far is has not been investigated, how important these pathways for the production of PHB during nitrogen starvation.

This work started with the initial aim to define whether PHB synthesis depends on the metabolism of glycogen. Since the initial results implied that PHB is strongly affected by glycogen catabolism, we further investigated the importance of the different carbon pathways EMP, ED and OPP for the production of PHB. These findings shall help to further understand the intracellular PHB metabolism in cyanobacteria, which can be used to create more efficient PHB overproduction strains, making the production of PHB as a bioplastic more cost efficient.

## 2. Results

Following the onset of nitrogen-starvation, large quantities of fixed carbon are stored in *Synechocystis* cells as glycogen granules. Long-term starvation experiments of *Synechocystis* cultures have shown that, while cells are chlorotic, glycogen is slowly degraded, following its initial rapid accumulation but PHB is slowly and steadily accumulating [9]. Considering that chlorotic cells are photosynthetically inactive, these data could indicate a potential correlation between the turn-over of glycogen and the synthesis of PHB. An overview of the metabolic pathways connecting the glycogen pool with PHB is shown in Figure 1. To substantiate the hypothesis that PHB might be derived from glycogen turn-over, we investigated PHB accumulation in various mutant strains, in which key steps in different pathways are interrupted. The respective mutations are shown in Figure 1. All strains used in this work were characterized previously, with their phenotypes, including growth behaviours, described in the respective publications (see Table A1). Furthermore, all mutants used in these studies were fully segregated to ensure clear phenotypes.

### 2.1. Impact of Glycogen Synthesis on PHB Production

**Figure 2:**
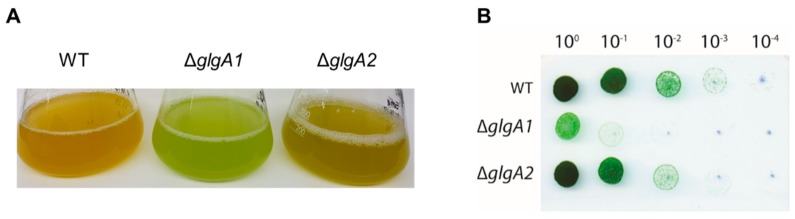
Characterization of the glycogen synthase mutants, ∆*glgA1* and ∆*glgA2*. (**A**) Cultures after five days of nitrogen starvation. (**B**) Recovery assay of chlorotic wild-type (WT) and mutants ∆*glgA1* and ∆*glgA2*, using the drop agar method. Cultures that were nitrogen-starved for 14 days were serially diluted from 1 to 1:10,000 and from each dilution, a drop of 5 µL was plated on BG11 agar and grown for seven days.

To analyze the role of glycogen synthesis on the production of PHB, we first analyzed the accumulation of these biopolymers during nitrogen starvation in mutants with defects in glycogen synthesis. The double mutant of the two glycogen synthase genes *glgA1* (*sll0945*) and *glgA2* (*sll1393*) is unable to acclimate to nitrogen deprivation and rapidly dies upon shifting cells to nitrogen free BG11^0^ medium [8] and, therefore, could not be analyzed. Instead, we used a knockout mutant of the glucose-1-phosphate adenylyltransferase (*glgC*, *slr1176*) and two knockout strains of each of the isoforms of the glycogen synthase, *glgA1* (*sll0945*) and *glgA2* (*sll1393*). Yoo et al. [28] reported that the single *glgA1* and *glgA2* mutants were still able to produce similar amounts of glycogen as the wild-type (WT), since one glycogen synthase is still present, and this seems to be sufficient to reach the wild-type levels of glycogen. However, the structure of the glycogen produced by the two isoforms seemed to slightly differ in chain-length distribution [28]. In that study, no distinguishing phenotype of the two mutant strains had been reported. In the present study, the cultures were shifted to nitrogen free medium BG11^0^ and further incubated under constant illumination of 40 µmol photons m^−2^ s^−1^. Under these experimental conditions, the ∆*glgA1* mutant showed an impaired chlorosis reaction, whereas the ∆*glgA2* mutant performed chlorosis as the wild-type strain (Figure 2A). To further determine the viability of two weeks nitrogen-starved cells, serial dilutions were dropped on nitrate-supplemented BG11 plates. As shown in Figure 2B, the ∆*glgA1* mutant was severely impaired in recovering from nitrogen starvation, whereas ∆*glgA2* could recover from chlorosis with the same efficiency as the wild-type (Figure 2B).

**Figure 3:**
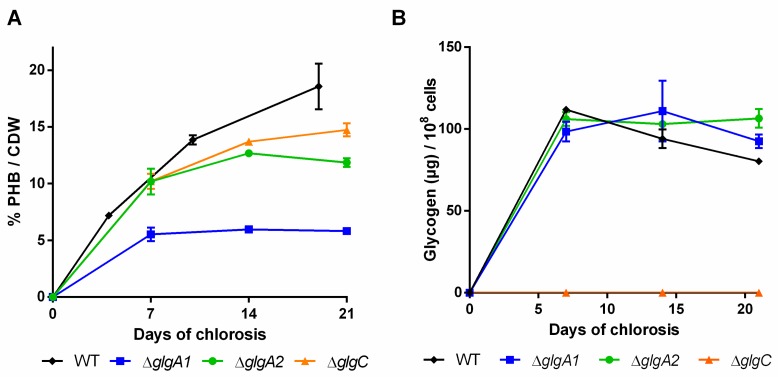
Polyhydroxybutyrate (PHB) content in percentage of cell dry weight (CDW) (**A**) and cellular glycogen content (**B**) of mutants impaired in the glycogen synthesis. Cultures were shifted to nitrogen free medium at day 0 and were subsequently grown for 21 days. Each point represents a mean of three independent biological replicates.

During the course of three weeks of nitrogen starvation, the quantities of PHB and glycogen that accumulate in the cells were determined (Figure 3A,B).

In the wild-type, the amount of glycogen already peaked after the first week and slowly decreased in the following two weeks (Figure 3B). As previously reported by Yoo et al. [28], the single ∆*glgA1* and ∆*glgA2* mutants initially accumulated similar amounts of glycogen to the wild-type, but in contrast to the wild-type, the level of glycogen remained high. The PHB content in the *glgA2* mutant was similar to the wild-type for the first seven days of nitrogen starvation, but PHB accumulation slowed down afterwards (Figure 3B). By contrast, the *glgA1* mutant was strongly impaired in PHB production. Together, the phenotype of the *glgA1* and *glgA2* mutants indicates that glycogen synthase GlgA1 plays a much more important role in nitrogen starvation acclimation than GlgA2, although the amount of glycogen produced by these two strains is almost the same. One explanation could be that the subtle differences in the glycogen produced form the two isoenzymes might result in different functions, with GlgA1-produced glycogen being much more relevant for the maintenance metabolism in chlorotic cells and for the resuscitation from chlorosis than glycogen produced by GlgA2. In clear correlation with the redundant role of GlgA2, the *glgA2* mutant was not impaired in PHB synthesis, whereas mutation of the functionally important *glgA1* gene resulted in strongly impaired PHB synthesis.

The *glgC* mutant was previously characterized by Grundel et al. [6]. They showed that ∆*glgC* is not able to perform a proper nitrogen-starvation acclimation response: it maintains its pigments while it loses viability, which was also observed in our experiments. Namakoshi et al. [29] showed that this mutant is unable to synthesize glycogen, which is also in line with our results. Under our conditions, unlike previously described by Damrow et al. [8], the *glgC* mutant did not show an increased amount of PHB compared to the WT, but seemed to accumulate less PHB instead (Figure 3A). It has to be noted though that Damrow et al. [8] investigated only one single timepoint, after seven days of nitrogen starvation. In addition, these results should be treated with care, since PHB content is normalized to cell dry weight, which shrinks in the *glgC* mutant due to progressive cell lysis. Consequently, the cell density was severely diminished at the end of the experiment (OD_750_ of 0.51, compared to ~1.15 of other mutants and the wild-type). The differences between our study and that of Damrow et al. [8] thus may result from differences in cell lysis rather than from differences in PHB synthesis. When the relatively low cell density of the *glgC* mutant is considered, it produces much less PHB per volume compared to the wild-type.

### 2.2. Impact of Glycogen Degradation on PHB Production

If glycogen turn-over would result in PHB accumulation during chlorosis, synthesis of PHB should be abrogated when glycogen degradation is impaired. To test this assumption, mutants in catabolic glycogen phosphorylase genes (*glgP*) were investigated with the same methods as described above. Glycogen can be degraded by the two glycogen phosphorylase isoenzymes, encoded by *glgP1* (slr1356) and *glgP2* (slr1367) [7]. A detailed study by Doello et al. [7] showed that GlgP2 is the main enzyme responsible for glycogen degradation during resuscitation from nitrogen chlorosis. Knocking out GlgP1 (Δ*glgP1*) does not affect the efficiency of recovery, whereas knocking out GlgP2 (Δ*glgP2*) or both phosphorylases (Δ*glgP1/2*) completely impairs the ability to degrade glycogen [7]. Here, we investigated glycogen and PHB accumulation during three weeks of nitrogen starvation in these glycogen phosphorylases mutants.

**Figure 4:**
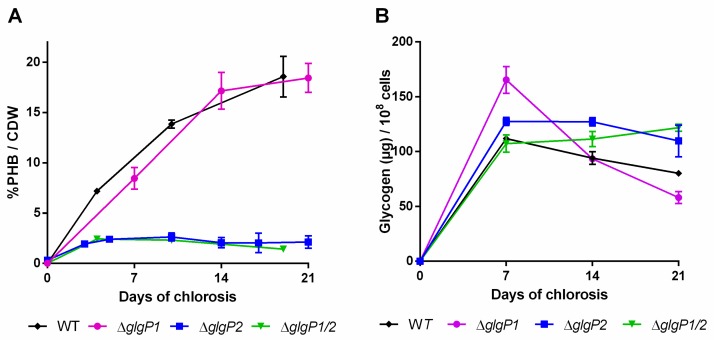
PHB content in percentage of cell dry weight (CDW) (**A**) and glycogen content (**B**) of mutants impaired in the glycogen degradation. Cultures were shifted to nitrogen free medium at day 0 and were subsequently grown for 21 days. Each point represents a mean of three independent biological replicates.

Although the initial amount of glycogen was higher in the *glgP1* mutant compared to the WT (Figure 4B), the amount decreased during the course of the experiment. By contrast, no glycogen degradation occurred in the ∆*glgP2* and ∆*glgP1/2* double mutant. This correlates with the specific requirement of chlorotic cells for GlgP2 for resuscitation from nitrogen starvation, as it has been previously described [7].

**Figure 5:**
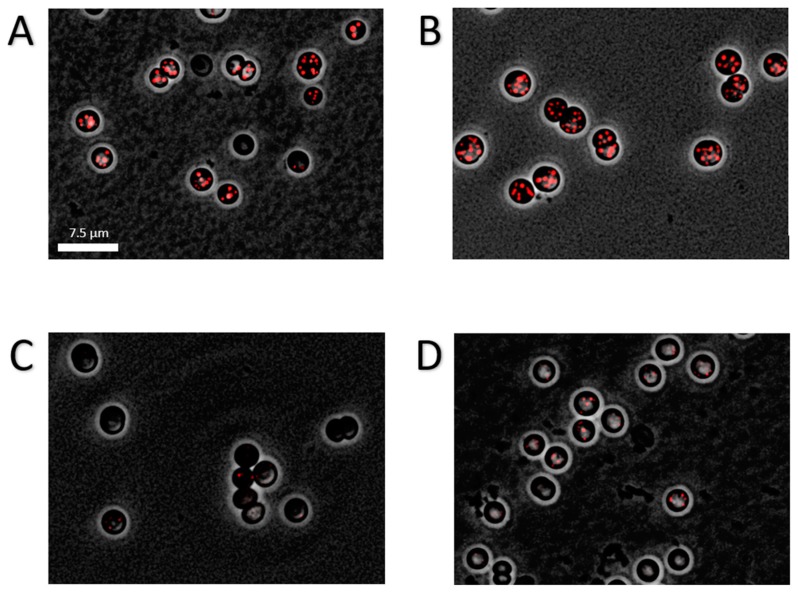
Fluorescence microscopic picture of Nile-red stained PHB granules in chlorotic cells. Cultures where grown for 14 days in nitrogen depleted medium BG11^0^. Shown is an overlay of phase contrast with a CY3 channel of the WT (**A**), ∆*glgP1* (**B**), ∆*glgP2* (**C**) and ∆*glgP1/2* (**D**). Scale bar corresponds to 7.5 µm.

Intriguingly, the different mutants showed a drastic difference in the amounts of PHB being produced (Figure 4A): While the ∆*glgP1* strain produced similar amounts of PHB as the wild-type, a strong decrease was observed for the ∆*glgP2* strain. The same phenotype was observed for the double knockout mutant, indicating that origin of the effect is based on the absence of *glgP2*. The PHB synthesis phenotypes were further confirmed by fluorescence microscopy after staining PHB with Nile red (Figure 5). PHB granules appear as bright red fluorescing intracellular granular structures. In agreement with the results from PHB quantification by HPLC analysis, the ∆*glgP1* strain showed similar amounts and distribution of PHB granules than the wild-type. By contrast, only very small granules, if at all visible, could be detected in the strains ∆*glgP2* and ∆*glgP1/2*.

Altogether, the inability of the mutants ∆*glgP2* and ∆*glgP1/2* to accumulate PHB demonstrates unequivocally that glycogen catabolism through GlgP2 is required for the ongoing PHB synthesis during prolonged nitrogen starvation.

### 2.3. Impact of Mutations in Carbon Catabolic Pathway on PHB Production

The experiments outlined above revealed that specific glycogen synthesizing or degrading enzymes have a strong effect on the amounts of PHB being produced and that glycogen turn-over via GlgP2 provides the carbon skeletons for PHB synthesis. To investigate how the released glucose phosphate molecules are metabolized downstream of glycogen, knockouts of the three most important glycolytic routes [25] were checked for their PHB and glycogen production during chlorosis. While the strain ∆*eda* (*sll0107*) lacks the ability to metabolize molecules via the ED pathway, ∆*gnd* is not able to use the OPP pathway. Additionally, the strain ∆*pfk1/2* lacks both phosphofructokinases, which causes an interruption of the EMP pathway. Also, the individual knockouts of both isoforms, ∆*pfk1* and ∆*pfk2*, were investigated.

**Figure 6:**
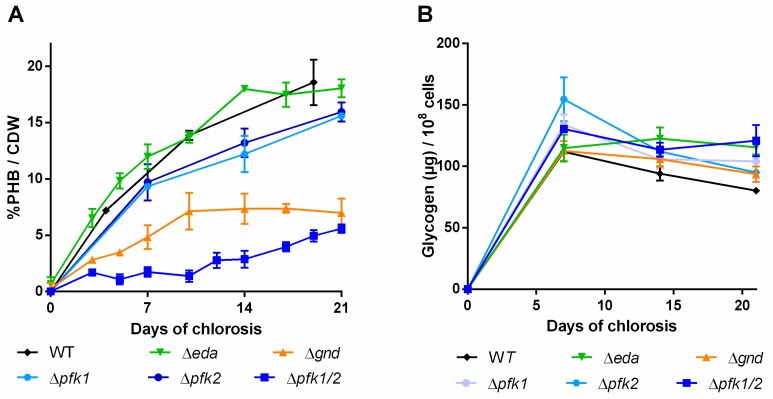
PHB content in percentage of cell dry weight (CDW) (**A**) and glycogen content (**B**) of mutants with disrupted carbon pathway. Cultures were shifted to nitrogen free medium at day 0 and were subsequently grown for 21 days. Each point represents a mean of three independent biological replicates.

Again, the different mutant strains and a WT control were grown for three weeks under nitrogen deprived conditions and PHB and glycogen content was quantified (Figure 6).

Distortion of the ED pathway (∆*eda*) did not result in any PHB phenotype different from the WT and the glycogen content remained high during the course of the experiment. In the ∆g*nd* mutant, a slower increase of PHB than in the WT was observed within the first ten days (Figure 6A) and PHB accumulation subsequently ceased. When the EMP pathway was blocked (∆*pfk1/2*), only very little PHB was produced in the first two weeks of the chlorosis. Thereafter though, PHB production slightly increased and finally reached similar levels as in the ∆*gnd* mutant. The total amount of glycogen over the time of chlorosis did not decrease in this mutant. The single ∆*pfk* mutants showed PHB contents similar to the WT, indicating that the two isoenzymes are able to replace each other’s function. Taken together, it appears that EMP and OPP pathways contribute to PHB production, whereas the ED pathway does not play a role.

### 2.4. Impact of PHB Formation on Glycogen Synthesis

**Figure 7:**
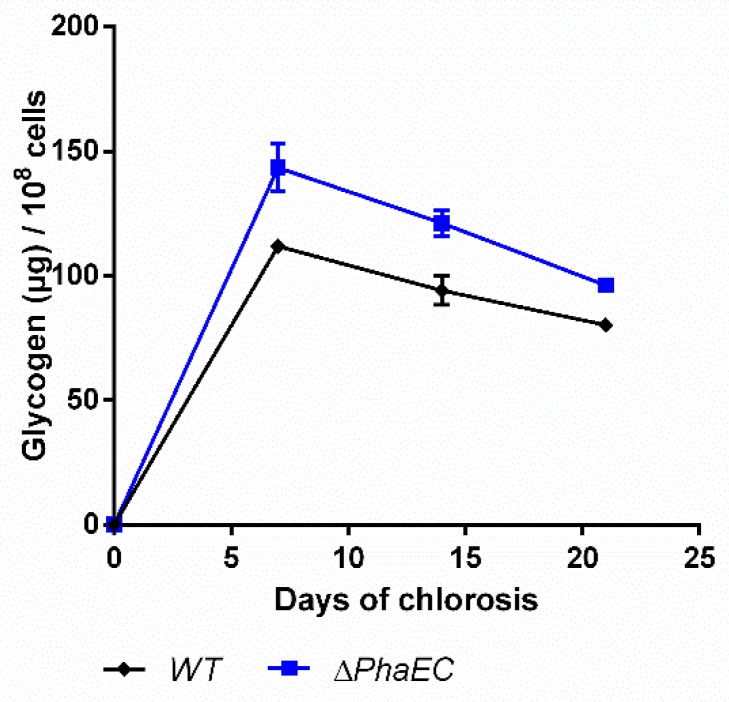
Glycogen content of wild-type and mutant lacking the PHB synthase genes (*PhaEC*). Cultures were shifted to nitrogen free medium at day 0 and were subsequently grown for 21 days. Each point represents a mean of three independent biological replicates.

In order to check how the PHB production affects the accumulation of glycogen, a PHB-free mutant, namely *∆phaEC*, was checked for its production of carbon polymers (Figure 7).

As expected, the mutant was unable to synthesize PHB (data not shown [8]). Compared to the WT, the mutant produced moderately higher amounts of glycogen and degrades it slightly faster, so that at the end of the experiments, the glycogen levels were quite similar.

## 3. Discussion

As recently shown by isotope labeling experiments [23], the majority of the carbon from PHB is coming from intracellular metabolites, which contribute around 74% to the carbon within PHB. Additionally, a random mutagenized strain, which is an overproducer of PHB, shows also a strongly accelerated decay of glycogen [30]. Here, we provide clear evidence that the intracellular glycogen pool and its products provide the carbon metabolites for PHB synthesis during nitrogen starvation. In the absence of glycogen degradation, as it is the case in the ∆*glgP2* and the ∆*glgP1/2* double mutant, PHB synthesis is almost completely abrogated. In the ∆*glgP2* mutant, the remaining GlgP1 enzyme is apparently not efficiently catabolizing glycogen, which agrees with its lack of function for the resuscitation from chlorosis. By contrast, GlgP2 is required for glycogen catabolism and resuscitation from chlorosis [7]. From these data, it is reasonable to hypothesize that during chlorosis, GlgP2 slowly degrades glycogen, and the degradation products end up in the PHB pool.

Like the two glycogen phosphorylase isoenzymes, the two glycogen synthase isoenzymes GlgA1 and GlgA2 appear to have specialized functions. Even though both ∆*glgA1* and ∆*glgA2* synthesized similar amounts of glycogen, deletion of *glgA1* resulted in a mutant with reduced bleaching, viability and PHB content whereas ∆*glgA2* was less affected. This indicates that glycogen produced by GlgA1 is important for resuscitation and growth. On the other hand, GlgA2 produced glycogen appeared less important or its function is yet unknown. Previous publications did not see such a difference, which may be explained by a much shorter time of nitrogen starvation used in their study [6]. Taken together, those mutants (*glgP2* and ∆*glgA1*) that were less viable under nitrogen starvation, did also synthesize less PHB. It remains to be demonstrated, if the different impact that GlgA1 and GlgA2 exert on viability and PHB synthesis originates from the slightly different branching patterns [28] of the glycogen, that they synthesize.

In the ∆*glgC* mutant, PHB is formed, although no glycogen is produced (Figure 3), which seems to contradict the hypothesis of glycogen-derived PHB [8,28]. Taking into account the above results, it appears likely that the carbon metabolites used for PHB can under certain conditions bypass the glycogen pool. When *glgC* is knocked out, glucose-1P (and its precursor, glucose-6P) cannot be further converted into ADP-glucose and may accumulate. The glucose-phosphates could then be downstream metabolized to glyceraldehyde-3P and further converted into PHB. By contrast, when *glgA1* is mutated, the newly fixed carbon can be converted by GlgC to ADP-glucose and subsequently enters the GlgA2-synthesized inactive glycogen pool, where it cannot be further metabolized into PHB. A similar connection between PHB and glycogen has already been described in other organisms (*Sinorhizobium meliloti*), where PHB levels were lower in a mutant lacking *glgA1* [31]. Since the GlgA1/GlgA2 double mutant rapidly dies upon nitrogen starvation [6], the impact of the complete absence of glycogen due to glycogen synthase deficiency on PHB synthesis cannot be experimentally tested.

Furthermore, we observed that a slower degradation of glycogen often correlates with a low-PHB-phenotype, as seen in the case of ∆*glgA1*, ∆*glgP2*, ∆*glgP1/2* and ∆*pfk1/2* (Figure 3, Figure 4 and Figure 6, respectively). This further supports the hypothesis, that only when glycogen gets degraded during the process of chlorosis, PHB is formed. In two cases, ∆*glgP1/2* and ∆*pfk1/2*, the amount of glycogen was even increasing during the later course of nitrogen starvation. This hints towards an ongoing glycogen formation during nitrogen chlorosis, which gets only visible once glycogen degradation is disturbed. Apparently, glycogen metabolism is much more dynamic than presumed from the relative static pool size observed in the wild-type. A steady glycogen synthesis may be counterbalanced by ongoing degradation, together resulting in only a slow net change of its pool size.

The residual metabolism in nitrogen starved chlorotic cells [9,32] is probably required to ensure long-term survival through repair of essential biomolecules such as proteins, DNA and RNA, osmoregulation, regulated shifts in metabolic pathways and the preparation for a quick response as soon as nutrients are available again [33,34]. According to these needs, non-growing starved cells still require a constant supply of ATP, reduction equivalents and the ability to produce cellular building blocks for survival. In line with this, we observed that PHB production was mainly achieved via the EMP pathway, which has the highest ATP yield among the three main carbon catalytic pathways (EMP, OPP, ED). In addition, we found that the OPP pathway is involved in PHB synthesis as well. This pathway provides metabolites for biosynthetic purposes as the repair of biomolecules for maintenance. By contrast, deletion of the ED pathway, which has a lower ATP yield in comparison to the EMP pathway and is physiologically probably most important in connection with photosynthesis and the Calvin–Benson cycle [7,25], did not impair PHB production under nitrogen starvation. Nevertheless, the glycogen levels did not decrease in the ∆*gnd* mutant, implying that mutation of the ED pathway affects the dynamics of glycogen turn-over discussed above.

Finally, we observed that the various carbon catabolic pathways have different functional importance for PHB production, in a time-dependent manner: While the mutant ∆*gnd* (blocking the OPP pathway) produced PHB in the first phase of the experiment but later stopped its synthesis, the ∆*pfk1/2* mutant (impaired in the EMP pathway) was initially blocked in PHB accumulation but later started to produce it (Figure 4). Interestingly, the time point, at which the ∆*gnd* mutant stopped PHB production matched its start in the ∆*pfk1/2* mutant. This could indicate a consecutive role of EMP and OPP pathway during nitrogen chlorosis. Although the exact function of the EMP pathway remains unknown, we show here for the first time a phenotype of a cyanobacterial mutant lacking this pathway. This suggested to also investigate the deletion of the individual knockouts. Deletion of only one of the Pfk isoenzymes (*pfk1*: sll1196 and *pfk2*: sll0745) resulted in mutants that produced about 80% of the PHB of WT cells, whereas the PHB production of the double mutant ∆*pfk1/2* was severely reduced. Pfk1 and Pfk2 can thus obviously compensate for the loss of the respective other, even though PHB production is highest if both enzymes are present. This observation is well in line with transcriptomic studies, which detected an increase in expression of both Pfk isoenzymes during nitrogen starvation [5]. The observation that both EMP and OPP pathway are of importance during arrested growth under nitrogen starvation is in agreement with earlier investigations that reported the upregulation of the sugar catabolic genes *pfk1*, *pfk2*, *zwf*, *gnd* and *gap1* concomitantly with glycogen accumulation [5,35]. EMP and OPP pathway thus support PHB production in non-growing, nitrogen-starved cells, whereas ED and OPP pathway are most important during resuscitation form nitrogen chlorosis after feeding the cells with nitrate [7].

The mutant ∆*phaEC* did not produce any PHB and degraded glycogen similarly to the WT (Figure 7). This indicates that there is no direct feedback between these two polymer pools. In the absence of PHB synthesis, metabolites form glycogen degradation could be leaked by overflow reactions. Under unbalanced metabolic situations, it has been shown that cyanobacteria can excrete metabolites into the medium to control their intracellular energy status [6,36,37]. In any case, this result demonstrates that PHB and glycogen do not compete for CO_2_ fixation products, but glycogen is epistatic over PHB synthesis.

Previous studies showed that, under nitrogen starvation, certain genes are upregulated, which are under the regulation of SigE, a group 2 σ factor [38]. Among these genes are glycogen degrading enzymes like *glgP1* and *glgP2* and *glgX*, but also the key enzymes for the pathways further downstream, namely *pfk* and *gnd.* The fact that all these genes are expressed simultaneously with the genes of the PHB synthesis [9], demonstrate that all relevant transcripts of the key enzymes required for the conversion from glycogen to PHB are present during nitrogen starvation. Our finding that PHB is mainly synthesized from glycogen degradation during nitrogen chlorosis is supported by a recent study, where a *Synechocystis* sp. PCC 6714 strain with enhanced PHB accumulation was created by random mutagenesis. Transcriptome analysis revealed that this strain exhibits an increased expression of glycogen phosphorylase [21]. This indicates that manipulation of glycogen metabolism may be a key for improved PHB synthesis.

Gaining further insights into the intracellular carbon fluxes could provide more information on how PHB production is regulated. Once the regulation is understood, this knowledge could be used to redirect the large quantities of glycogen towards PHB. This knowledge could be used in metabolic engineering approaches to either completely reroute the carbon from glycogen (making up more than 60% of the CDW) to PHB, for example by overexpression of glycogen degrading enzymes, or even from inorganic carbon to PHB directly. Therefore, the new insights from this work can be exploited for biotechnological applications to further increase the amounts of PHB being produced in cyanobacteria.

## 4. Materials and Methods

### 4.1. Cyanobacterial Cultivation Conditions

For standard cultivation, *Synechocystis* sp. PCC 6803 cells were grown in 200 mL BG_11_ medium, supplemented with 5 mM NaHCO_3_ [39]. A list of the used strains of this study is provided in Table A1. Two different wild-type strains, a Glc sensitive and a Glc tolerant one, were used. Both strains showed the same behavior during normal growth as well as during chlorosis. Appropriate antibiotics were added to the different mutants to ensure the continuity of the mutation. The cells were cultivated at 28 °C, shaking at 120 rpm and constant illumination of 40–50 μmol photons m^−2^ s^−1^. Nitrogen starvation was induced as described previously [40]. In short, exponentially growing cells (OD 0.4–0.8) were centrifuged for 10 min at 4000× *g*. The cells were washed in 100 mL of BG_0_ (BG_11_ medium without NaNO_3_) before they were centrifuged again. The resulting pellet was resuspended in BG_0_ until it reached an OD of 0.4.

### 4.2. Microscopy and Staining Procedures

To observe PHB granules within the cells, 100 µL of cyanobacterial cells were centrifuged (1 min at 10,000× *g*) and 80 µL of the supernatant discarded. Nile Red (10 µL) was added and used to resuspend the pellet in the remaining 20 µL of the supernatant. From these mixtures, 10 µL were taken and applied on an agarose coated microscope slide to immobilize the cells. The Leica DM5500B microscope (Leica, Wetzlar, Germany) was used with a 100× /1.3 oil objective for fluorescence microscopy. To detect Nile red stained PHB granules, an excitation filter BP 535/50 was used, together with a suppression filter BP 610/75. A Leica DFC360FX (Leica, Wetzlar, Germany)) was used for image acquisition.

### 4.3. PHB Quantification

PHB content within the cells was determined as described previously [41]. Roughly 15 mL of cells were harvested and centrifuged at 4000× *g* for 10 min at 25 °C. The resulting pellet was dried for 3 h at 60 °C in a speed-vac (Christ, Osterode, Germany), before 1 mL of concentrated H_2_SO_4_ was added and boiled for 1 h at 100 °C to break up the cells and to convert PHB to crotonic acid. From this, 100 µL were taken and diluted in 900 µL 0.014 M H_2_SO_4_. To remove cell debris, the samples were centrifuged for 10 min at 10,000× *g*, before 500 µL of the supernatant were transferred to 500 µL 0.014 M H_2_SO_4_. After an additional centrifugation step with the same conditions as above, the supernatant was used for HPLC analysis on a Nucleosil 100 C 18 column (Agilent, Santa Clara, CA, USA) (125 by 3 mm). As a liquid phase, 20 mM phosphate buffer (pH 2.5) was used. Commercially available crotonic acids was used as a standard with a conversion ratio of 0.893. The amount of crotonic acid was detected at 250 nm.

### 4.4. Glycogen Quantification

Intracellular glycogen content was measured by harvesting 2 mL of cyanobacterial culture. The cells were washed twice with 1 mL of ddH_2_O. Afterwards the pellet was resuspended in 400 µL KOH (30% *w/v*) and incubated for 2 h at 95 °C. For the subsequent glycogen precipitation, 1200 µL ice cold ethanol (final concentration of 70%) were added. The mixture was incubated at –20 °C for 2–24 h. Next, the solution was centrifuged at 4 °C for 10 min at 10,000× *g*. The pellet was washed twice with 70% and 98% ethanol and dried in a speed-vac for 20 min at 60 °C. Next, the pellet was resuspended in 1 mL of 100 mM sodium acetate (pH 4.5) and 8 µL of an amyloglucosidase solution (4.4 U/µL) were added. For the enzymatic digest, the cells were incubated at 60 °C for 2 h. For the spectrometical glycogen determination, 200 µL of the digested mixture was used and added to 1 mL of O-toluidine-reagent (6% O-toluidine in 100% acetic acid). The tubes were incubated for 10 min at 100 °C. The samples were cooled down on ice for 3 min, before the OD_635_ was measured. The final result was normalized to the cell density at OD_750_, where OD_750_ = 1 represents 10^8^ cells. A glucose standard curve was used to calculate the glucose contents in the sample from their OD540.

### 4.5. Drop Agar Method

Serial dilutions of chlorotic cultures were prepared (10^0^, 10^−1^, 10^−2^, 10^−3^, 10^−4^ and 10^−5^) starting with an OD_750_ of 1. Five microliters of these dilutions were dropped on solid BG_11_ agar plates and cultivated at 50 μmol photons m^−2^ s^−1^ and 27 °C for 7 days.

## Appendix A

**Table A1:** List of used strains.

Strain	Background	Relevant Marker of Genotype	Reference
*Synechocystis* sp. PCC 6803 GS	GS	-	Pasteur culture collection
*Synechocystis* sp. PCC 6803 GT	GT	-	Chen et al. 2016
Δ*glgA1*	GT	*sll0945::kmR*	Gründel et al. 2012
Δ*glgA2*	GT	*sll1393::cmR*	Gründel et al. 2012
Δ*glgC*	GT	*slr1176::cmR*	Damrow et al. 2012
Δ*glgP1*	GS	*sll1356::kmR*	Doello et al. 2018
Δ*glgP2*	GS	*slr1367::spR*	Doello et al. 2018
Δ*glgP1/2*	GS	*sll1356::kmR*, *slr1367::spR*	Doello et al. 2018
Δ*eda*	GT	*sll0107::gmR*	Chen et al. 2016
Δ*gnd*	GT	*sll0329::gmR*	Chen et al. 2016
Δ*pfkB1/2*	GT	*sll1196::kmR*, *sll0745::spR*	Chen et al. 2016
Δ*phaEC*	GS	*slr1829*, *slr1830::kmR*	Klotz et al. 2016
